# Evaluating the Mechanical and Tribological Properties of 3D Printed Polylactic-Acid (PLA) Green-Composite for Artificial Implant: Hip Joint Case Study

**DOI:** 10.3390/polym14235299

**Published:** 2022-12-04

**Authors:** Ahmed Fouly, Abdulaziz K. Assaifan, Ibrahim A. Alnaser, Omar A. Hussein, Hany S. Abdo

**Affiliations:** 1 Department of Production Engineering and Mechanical Design, Faculty of Engineering, Minia University, Minia 61519, Egypt; 2 Mechanical Engineering Department, College of Engineering, King Saud University, Riyadh 11421, Saudi Arabia; 3 King Abdullah Institute for Nanotechnology, King Saud University, P.O. Box 2455, Riyadh 11451, Saudi Arabia; 4 Biomedical Technology Department, College of Applied Medical Sciences, King Saud University, Riyadh 12372, Saudi Arabia; 5 Center of Excellence for Research in Engineering Materials (CEREM), King Saud University, P.O. Box 800, Riyadh 11421, Saudi Arabia; 6 Mechanical Design and Materials Department, Faculty of Energy Engineering, Aswan University, Aswan 81521, Egypt

**Keywords:** PLA composite, hip joint replacement, date pits filler, 3D printing, fused filament fabrication

## Abstract

Artificial implants are very essential for the disabled as they are utilized for bone and joint function in orthopedics. However, materials used in such implants suffer from restricted mechanical and tribological properties besides the difficulty of using such materials with complex structures. The current study works on developing a new polymer green composite that can be used for artificial implants and allow design flexibility through its usage with 3D printing technology. Therefore, a natural filler extracted from corn cob (CC) was prepared, mixed homogeneously with the Polylactic-acid (PLA), and passed through a complete process to produce a green composite filament suit 3D printer. The corn cob particles were incorporated with PLA with different weight fractions zero, 5%, 10%, 15%, and 20%. The physical, mechanical, and tribological properties of the PLA-CC composites were evaluated. 3D finite element models were constructed to evaluate the PLA-CC composites performance on a real condition implant, hip joints, and through the frictional process. Incorporating corn cob inside PLA revealed an enhancement in the hardness (10%), stiffness (6%), compression ultimate strength (12%), and wear resistance (150%) of the proposed PLA-CC composite. The finite element results of both models proved an enhancement in the load-carrying capacity of the composite. The finite element results came in line with the experimental results.

## 1. Introduction

With age, some joints and parts of the human body start to wear out, which can cause difficulty in movement and a lot of pain. To support and help the elderly in such cases, some researchers tried to design exoskeletons that can help them in daily work [1]. However, orthopedic doctors work on replacing deteriorated joints and performing restoration operations for some bony regions [2]. In such surgeries, there are two main problems; first, the design complexity of such parts, which is reflected in the difficulty of the production process using available materials in the market like ceramics, metals, and polymers [3]. The second problem is the possibility of the utilized materials to withstand different loading conditions and prevent any postoperative problems. Polymers provide many advantages that many products may need, such as ease of preparation, lightweight, and corrosion resistance, in addition to the fact that many polymers are considered biomedical materials [4]. However, the specifications of polymeric materials are poor for dealing with different types of loads. Also, the available production processes used with polymers may prevent carrying out complex designs as required. Hence, the challenges researchers face are developing new production methods that can deal with complex designs and enhancing the mechanical and tribological properties of the utilized polymeric materials.

Nowadays, additive manufacturing technique has acquired academic and industrial interest due to its conspicuous ability to generate complex structures. The main working mechanism of the additive manufacturing technique is translating a 3D (computer aided design) CAD model into 3D solid parts by laying down layers of polymeric materials [5]. The additive manufacturing technique is widespread and is anticipated to widen in the future due to its use in different fields like aerospace, healthcare, automotive, education, and construction. The additive manufacturing technique is a general expression that includes many sub-techniques like selective laser sintering, fused filament fabrication, stereolithography, selective laser melting, and 3D printing [6,7]. Nevertheless, extrusion-based additive manufacturing techniques like fused filament fabrication have gained tremendous industrial attentiveness. In fused filament fabrication, a polymeric material comes in the shape of a filament with a standard diameter. The filament pass-through a heated nozzle for melting before deposition as successive layers on the surface of a platform. The deposited layers get adhered to each other and solidify, forming the 3D part in its final shape. Although fused filament fabrication (3D printing) is considered a straightforward process, it has some limitations and shortages [8]. Warping and the poor mechanical properties of the 3D-printed parts are the most prevalent troubles that restrict polymer type proper for 3D printing technique [9].

Among the different types of biopolymers, Polylactic acid (PLA) is very famous in use for fused filament fabrication 3D-printing. PLA is characterized by its minimum warping issues, biodegradability, environmentally friendly, renewable, environmentally friendly, and ease of preparation and use. There are two main methods for PLA production: ring-opening polymerization of lactide and direct polycondensation of lactic acid that is extracted from renewable resources like cassava roots, corn starch, and sugarcane [10]. Compared with different synthetic polymeric materials, PLA has good strength and stiffness, allowing industries to use it in various applications like medical applications, automotive, and packaging [11]. However, the main limitation of PLA when used in 3D printing technology is the inferiority of the printed parts mechanical and tribological properties when compared with other parts fabricated by conventional processes like injection molding and compression molding.

The shortage in the mechanical and tribological properties of PLA could be addressed to an acceptable level by modifying it using the methodology of composites [12]. Many investigations dealt with PLA composites and validated their results by 3D printing demonstrated samples. Patanwala et al. [13] investigated the effect of reinforcing PLA with different loading fractions, 0.5 to 5 wt.%, of carbon nanotube on its mechanical properties. They recorded an enhancement in Young’s modulus, reaching 30% for 5 wt.% CNT. However, the toughness and tensile strength have deteriorated. Heidari-Rarani et al. [14] studied the effect of adding carbon fiber to the PLA on the mechanical properties of the PLA. The results showed an outstanding enhancement in the bending and tensile characteristics of the PLA. Ertane et al. [15] investigated the tribological performance of PLA/Biogenic carbon composites. Results showed stability in the friction when the biogenic carbon volume fraction reached 30%, with a noticeable increase in wear resistance. Growing anxiety towards using reinforcement materials that may be toxic when used with biomedical applications led researchers to develop new materials that are safe, biomedical, and environment-friendly. Natural materials reinforced polymer composites attracted the attention of many researchers due to their safety in use with biomedical applications (biocompatibility) [16]. Natural reinforcement can be prepared based on simple processes applied to natural materials available in nature. The research is in progress towards enhancing the mechanical and tribological properties of natural materials reinforced polymers. More than 90% of failures in many applications occurred because of the polymeric material strength and the tribological loading situation [17]. Fouly et al. [18] evaluated the mechanical characteristics of 3D printed PLA reinforced with micro date pits powder with different weight fractions to be used to fabricate artificial hip joints. Results showed an enhancement in the overall mechanical properties. In the same way, Tisserat et al. [19] tried to improve the properties of PLA using two different types of wood, Paulownia and Osage orange woods. The authors found an increase in the elasticity and elongation accompanied by a decrease in the tensile strength. Utilizing wood, another study reinforced PLA with beechwood with weight fraction up to 50% and investigated the composite’s rheological and mechanical properties [20]. They noticed a deterioration in the storage modulus but the the glass transition temperature was the same. Furthermore, the roughness of the samples increased with the increase of beechwood loading fraction with the appearance of some wood clusters and voids on the samples surface. Liu et al. [21] tried to estimate the change in the mechanical properties of PLA due to the addition of various types of natural fillers, pulp, lignin, eucalyptus, newspaper, and pine. Results showed a 74% enhancement in the tensile strength with the incorporation of 15 wt.% lignin. Kanakannavar et al. [22] investigated the friction coefficient and specific wear rate of PLA reinforced with natural fibre 3D braided woven fabric. They found that incorporation of natural fibre 3D braided woven fabric into the PLA enhanced the samples surface roughness considerably. When the reinforcement weight rachio reached 35%, the highest wear resistance was recorded.

Agricultural Waste is a natural material that remains from fruits and vegetables; it is unusable and unwanted materials like fruit, trees, and grape vines. Corn cob is one of the most popular agricultural wastes in many countries. After utilizing corn for different purposes, the remaining corn cob becomes a waste and has no use. Consequently, many researchers tried to transfer corn cob from just waste into useful materials, filler, that can be used in biocomposites. Chen et al. [23] tried to convert the corn cob into fibers; then, they investigated the effect of incorporating corn cob powder as a filler into polyethylene on the mechanical properties of the produced composite. The results showed an enhancement in both mechanical and weathering behavior. Another team worked on converting corn cob to a micro filler and used it with different loading fractions as a reinforcement to epoxy [24]. At 8 wt.% corn cob, results showed an enhancement in the compressive yield strength and Young’s modulus by 22.22% and 21.26%, respectively. Furthermore, the enhancement in mechanical properties is accompanied by an improvement in wear resistance. Zhu et al. [25] evaluated the mechanical characteristics and water absorption of high-density polyethylene reinforced by corn cob powder. They found that incorporating corn cob into high-density polyethylene enhanced the flexural moduli and strengths by up to 40%. In addition, an increase in the water absorption of the composite is accompanied by an increase in corn cob weight fraction. Another study investigated the effect of reinforcing Polymethyl methacrylate (PMMA) by corn cob on the mechanical and tribological performance of composites used in dentures [26]. Results showed an enhancement in the mechanical and tribological characteristics of the PMMA composites which in turn can enhance the quality of the dentures.

Based on the previous survey, it is obvious that the optimal choice to achieve complex designs for biomedical applications is using fused filament fabrication (3D printing). Furthermore, utilizing natural materials as a filler is able to enhance the different properties of composites. Consequently, the current study evaluates the effect of reinforcing PLA with different loading fractions of corn cob, 5, 10, 15, and 20 wt.%. There is no standard method for converting corn cob from its original nature to a useful filler. Moreover, there is no available data regarding the usage of the produced corn cob filler in composites without purification treatment. The current study conducted a complete production process for the corn cob filler and the PLA/corn cob filament used in 3D printers. The mechanical and tribological properties of the produced composite with different loading fractions of corn cob were investigated. As a case study, the experimental results were fed into ANSYS, and a finite element model for a hip joint was constructed based on the experimental results. The load-carrying capacity of the produced composites was evaluated based on finite element results.

## 2. Materials and Experimental Work

### 2.1. Materials and Samples Preparation

PLA matrix was purchased from Shanghai Nuolei CNC Router Equipment Co., Ltd., Shanghai, China. According to the datasheet, it is 100 new material made of corn starch with no impurity and minimal warping and shrinkage. It was purchased as a filament with 1.25 g/cm^3^ density, 1.75 mm diameter, and the printing temperature varies between 190 °C up to 220 °C. Corn is classified as a fruit by botanists, and corn cob is the backbone of such a fruit where the corn kernels emerge around it. To transfer the corn cob from its original nature to a filler powder, the corn kernels were removed, and remained corn cobs were exposed to the sun rays for three months to remove moisture. Thereafter, using a hummer, the corn cobs were crashed into smaller parts and smashed again into a fine powder using a grain miller for 3 h at a speed of 250 rpm. The final corn cob powder was exposed to 70 °C inside an oven for 5 h to remove any remained moisture.

**Figure 1:**
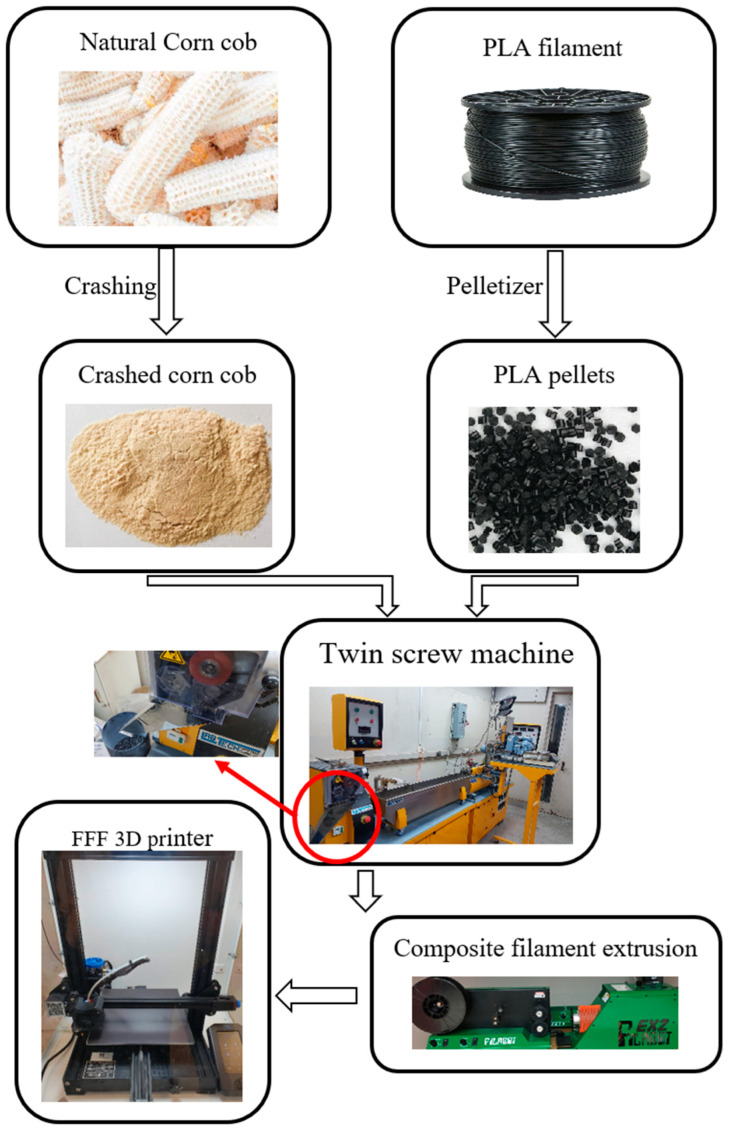
Flowchart of PLA-corn cob composite filament production and the fabrication of test specimens using an FDM 3D printer.

To prepare the PLA/corn cob composite sample, the PLA filament was cut into pellets using a pelletizer. The corn cob powder was weighted and mixed ultrasonically with ethanol to prohibit powder agglomeration. After weight, the PLA pellets were added to the corn cob powder and mixed mechanically using a mechanical stirrer for 30 min at 300 rpm to disperse the corn cob powder into the PLA and allow ethanol to evaporate. The PLA/corn cob composite was prepared with different corn cob weight fractions 0, 5, 10, 15, and 20 wt.%, and they are referred to as PLA-CC0, PLA-CC5, PLA-CC10, PLA-CC15, and PLA-CC20, respectively. The PLA-CC mixture was set into a vacuum oven at a temperature of 50 °C to remove any remaining ethanol for 24 h. Then, the PLA-CC mixture was inserted in a twin-extruder machine and extruded to create the composite filaments. The twin-extruder machine has ten zones of various temperatures varying from 160 °C up to 210 °C, and the running speed is 50 rpm. The screw length-to-diameter ratio is 40, with a diameter of 16 mm. After the mixture gets out of the extruder nozzle, the PLA-CC filament is cooled inside a water path and cut again into pellets through the pelletizer. To ensure homogenous composite filament, the composite pellets were taken again into the twin-extruder machine, and the process was reiterated three times for each PLA-CC composition. The mixing process was conducted without any binding assistive approach or any treatment materials. To produce a composite filament that can be used with 3D printer, the PLA-CC composite pellets were taken to a single screw extruder model Filabot EX2. The tested samples were created on CAD software and imported to a software, Cura, to generate the G-code for the 3D printer. Then, the specimens were created on the 3D printer model; Creality 3D^®^ Ender-3 V2, Shenzhen Creality 3D Technology Co., Ltd., Shenzhen, China. The 3D printing setting van considerably affects the different properties of the 3D printed parts. Consequently, to maintain the variation of CC weight fraction in the current study as the only variable, the 3D printer parameters were fixed during the printing of all PLA-CC composites; nozzle temperature 200–210 °C, bed temperature 70 °C, and density of the 3D printed samples 100%. Figure 1 illustrates the PLA-CC composite sample production process.

### 2.2. Characterization and Testing

The crystallinity of corn cob powder, PLA, and PLA-CC composites were evaluated using an X-Ray diffraction pattern (XRD) that uses D8 discover from Bruker, Germany. The morphology of the produced powder was observed using field emission scanning electron microscopy; model: JEOL JSM-7600F, Tokyo, Japan. The crystallinity of the produced PLA-DP composites was evaluated using an X-Ray diffraction pattern (XRD). Furthermore, Thermal decomposition patterns of the PLA-DP composites were performed using thermogravimetric TGA on the TA-Q500 System of TA. Approximately 10 mg of each PLA-DP composite sample was heated with a heating rate of 10 °C/min from 30 to 800 °C, under a nitrogen atmosphere utilizing TG-DTA: NETZSCH Germany (Model: STA 449 F3).

One of the most important parameters representing the composite’s physical properties is density. The density of the composite usually depends on the relative ratio between the matrix and filler. According to Archimedes’ principle [27], the densities of PLA-CC composites were examined experimentally. The PLA-CC composite samples were weighted in air and alcohol. The densities were calculated as shown in Equation (1). Where *ρ*_PLA-CC_, *ρ*_alc_, and *ρ*_air_ are the densities [g/cm^3^] of the PLA-CC composites, alcohol, and air, respectively; *m_air_* and *m_alc_* are the masses of the PLA-CC composites [g] in air and alcohol, respectively. The density for each PLA-CC composite specimen was measured 5 times, and the average was calculated considering the standard errors.

(1) $$\rho_{PLA-CC}=\left(\rho_{alc}-\rho_{air}\right)\times \frac{m_{air}}{m_{air}-m_{alc}}+\rho_{air}$$

The mechanical properties of PLA-CC composite samples were identified using hardness and compression tests. The Shore D Durometer measured the hardness based on ASTM D2240 [28], where the load capacity is 5 ± 0.5 kg and the measuring time is 15 s. To ensure hardness stability along the samples surfaces, the hardness was tested on different locations on the surfaces of the sample, and the average was calculated. To apply the compression test on the PLA-CC samples, samples were prepared with a height of 16 mm and a diameter 8 mm based on ISO 604 Plastics [29]. The compression test was conducted on an Instron 5582 Micro-tester (Instron, University Ave, Norwood, MA, USA), and the compression rate was set at 2 mm/min. Based on the stress-strain diagram of each sample, ultimate compressive strength, Young’s modulus, elongation, and toughness were calculated.

**Figure 2:**
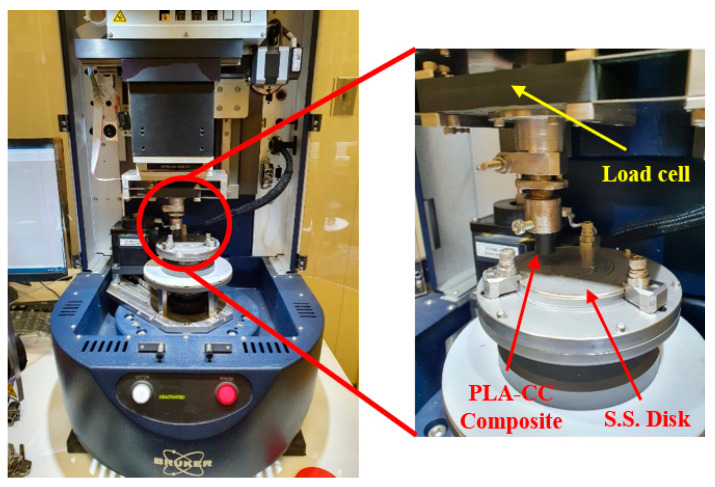
The tribometer for tribological test.

The friction coefficient and specific wear rate for PLA-CC samples were specified using the pin-on-disc test and dry sliding results, according to ASTM G99-95, universal tribometer Mod. UMT-2MT testing block sin T45815 Bruker-Nano Surfaces, as shown in Figure 2. The tribological tests were conducted at a speed of 0.4 m/s, humidity of 60%, and temperature of 26 °C. The PLA-CC composite sample dimensions are 20 mm in length and 8 mm in diameter. The counter face (disc) is stainless steel with a diameter of 8 cm and a surface roughness of 12.5 µm. After each test, the stainless-steel disk was cleaned with acetone and dried using a heat gun to prevent contaminants from other experiments. The tribological results were recorded under loads of 5, 10, 15, and 20 N. In addition, the effect of the sliding time was considered 5, 10, 15, and 20 min. The tribological test was performed six times for each PLA-CC composite sample, and the average coefficient of friction was estimated. The mass of each sample was measured before and after the tribological test, and the specific wear rate was calculated according to Equation (2).

(2) $$\mathrm{Specific}\;\mathrm{wear}\;\mathrm{rate}=\frac{\Delta m}{\rho \;P_{n}\;L}$$

where Δ*m* is the difference in mass before and after the tribological test; *ρ* is the density of each PLA-CC composite sample; *P_n_* is the normal load, and *L* is the frictional distance.

### 2.3. Finite Element Analysis (FEA)

#### 2.3.1. Case Study: Hip Joint

**Figure 3:**
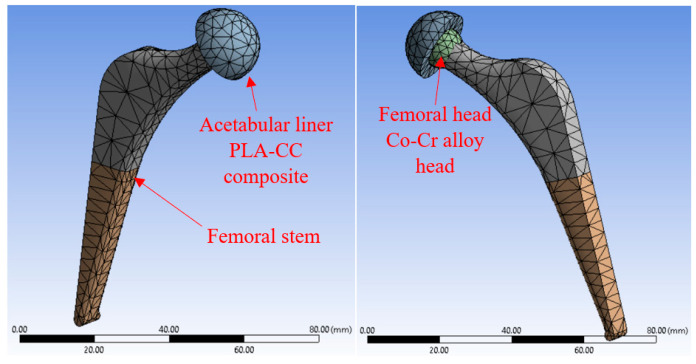
The finite element model of the hip joint.

The hip joint was selected as a case study to evaluate the performance of the produced PLA-CC composites on a real biomedical application. In the hip joint, whatever the materials are used in manufacturing, a part called an acetabular liner is made of polymer to prevent direct friction between metallic or ceramic components. The acetabular liner is the part that is usually exposed to different loads due to the motion of the human. Consequently, a finite element model for the hip joint is built using ANSYS, as shown in Figure 3, to investigate the effect of such loads on the performance of the proposed PLA-CC composites. Based on a previous hip joint model that was initiated in JAPAN, the model of the hip joints consists of two main parts [30]. A ball representing the femoral head is usually made of ceramic or Co-Cr alloy; and the acetabular liner is made of polymeric material. The model was meshed automatically by the ANSYS, which meshed the model into hexahedron and tetrahedron elements. After meshing, the model has 2525 nodes and 1247 elements. The boundary conditions were defined according to a previous study where the human load was defined in three directions on the acetabular liner of the hip joint [31]. Based on this study, the forces arising from stumbling for a 100 kg human weight are 1540, 3480, and 70 N in x, y, and z, respectively. Moreover, the ball of the femoral stem is fixed in three directions. To evaluate the performance of the joint in harsh conditions, the contact between the joint parts is defined as bonded. Based on the mechanical properties of the PLA-CC composites, the material of the acetabular liner is defined in the model. After running the model, the maximum shear stress and equivalent stress were recorded.

#### 2.3.2. FEA of the Frictional Process

**Figure 4:**
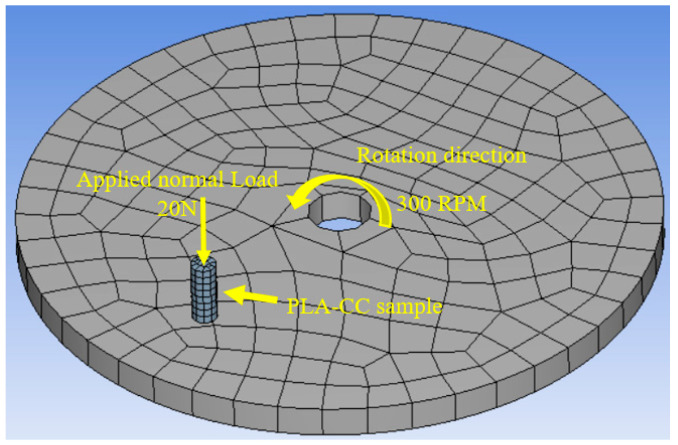
The finite element model of the frictional process.

To validate the load-carrying capacity of the PLA-CC composites based on the frictional process, the contact stress during the frictional process should be evaluated. Therefore, an FEA model was built that represents the tribological test using the bin on disk using ANSYS software, as shown in Figure 4.

To measure the different stresses during the frictional process, the contact between the pin and the disk was defined as frictional, and the solution is based on Lagrange contact. The diameter of the disk was set to 20 cm, while the PLA-CC pin was 20 mm in length and 8 mm in diameter. The ANSYS automatic mech made the elements a mix between hexahedrons and tetrahedrons with 325 elements and 2260 nodes. The PLA-CC sample was subjected to 20 N normal load in the z direction; however, it was fixed in x and directions. The PLA-CC properties were defined based on the experimental results. The stainless-steel disk was rotating at a fixed speed of 300 rpm.

## 3. Results and Discussion

**Figure 5:**
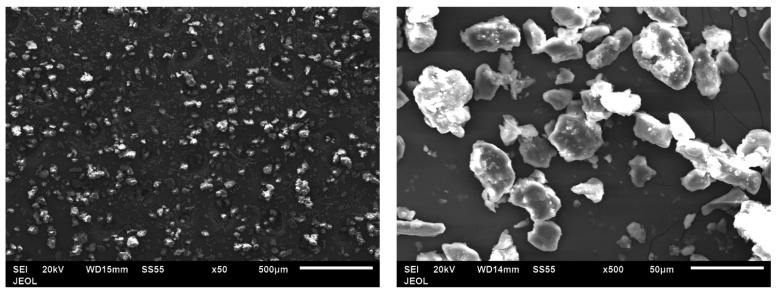
SEM image of grain milled corn cob particles.

The grain-milled corn cob morphology was identified using scanning electron microscope, as shown in Figure 5. It is clear that the corn cob particles are non-uniform in shape and the particle size varies between a few microns to 30 microns. The surface of corn cob particles is rough, and there is a deposition of very fine corn cob particles on the surface of larger ones.

**Figure 6:**
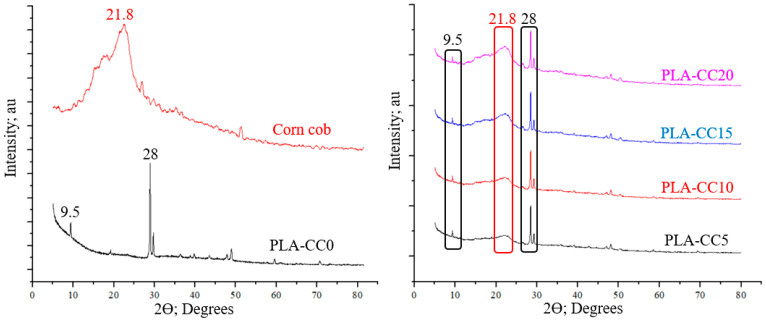
XRD patterns of PLA, Corn cob, and PLA-CC composites.

To evaluate the quality of the PLA-CC produced composite. The effect of corn cob insertion on the chemical composition of the PLA, X-ray diffraction (XRD) analysis was performed for PLA, corn cob, and each produced PLA-CC composite, as shown in Figure 6. The XRD showed two main peaks for the pure PLA and one main peak for the corn cob. The high-intensity peak of PLA appears at 2θ of 28.5°; however, the low-intensity one at 2θ of 9.5° which is the same XRD results achieved by Kumar et al. [32]. On the other hand, the main peak of corn cob appeared at 2θ of 21.8°, which is the main peak of alpha-cellulose, as mentioned by Yu et al. [33]. The PLA peak that appeared at 9.5° indicates the addition of cereal starch during the production of the PLA, which in turn raises the PLA biodegradability [30]. In addition, the appearance of the two peaks at 9.5° and 21.8° signifies the extraction of cereal starch from wheat, potato, and tapioca [34]. The pyramid shape of the corn cob XRD pattern indicates the amorphous nature of the corn cob. The XRD pattern of the PLA-CC composites shows a combination between the main peaks of the PLA and the corn cob. However, the intensity of the corn cob peaks varies according to the corn cob weight fraction inside the PLA, which in turn reflects the homogenous distribution of corn cob particles inside the PLA. The absence of new peaks in the XRD pattern of the PLA-CC cob composite represent an indication of the quality of the composite, where no chemical reaction occurred between the PLA and the corn cob [35]. Furthermore, such a composite pattern illustrates that the incorporation of corn cob inside the PLA didn’t affect its original structure.

**Figure 7:**
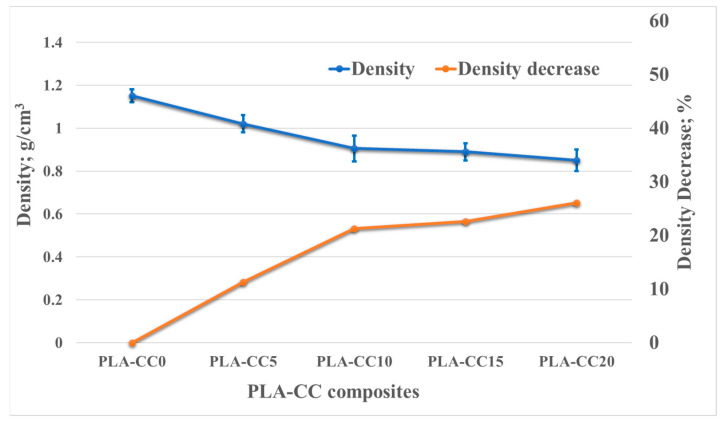
PLA-CC composites densities for different corn cob weight fractions.

Density as a physical property is considered one of the most important issues when dealing with composites. The light weight of polymers is one of the merits that encourage researchers and industrials to use polymers in different applications. Adding filler to enhance the mechanical properties of polymers may positively or negatively affect their lightweight, which directly depends on the density. Consequently, in the current study, the densities of the PLA-CC composite were measured experimentally. The density of composites counts on the density of both PLA and corn cob. Therefore, Figure 7 shows a dependency and change in the composite density accompanied by the corn cob weight fraction change. It is obvious that incorporating corn cob inside the PLA decreases it density of the PLA-CC composite. Furthermore, increasing the corn cob weight fraction led to more decrease in density, reaching 26% for 20 wt.% corn cob. The continuous decrease in the density accompanied by the increase of corn cob weight fraction indicates that the density of corn cob filler is lower than the PLA. Consequently, the incorporation of corn cob weight fraction with low density replacing the weight fraction of high-density PLA led to a decrease in the overall density of the composite. This could be an added advantage in that the composite becomes lighter.

**Figure 8:**
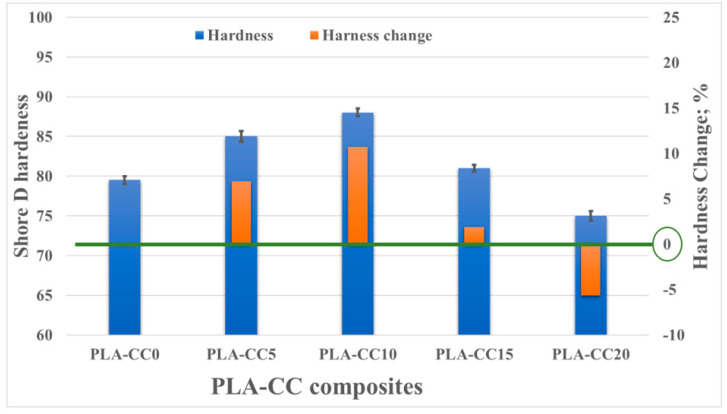
PLA-CC composites hardness for different corn cob weight fractions.

Figure 8 shows the change in the shore D hardness of the PLA-CC composite with the change in corn cob weight fraction. The hardness of the composite increased up to 10%, compared with pure PLA, when the weight fraction of corn cob was 10 wt.%. However, increasing the weight fraction by more than 10% led to a deterioration in the hardness of the composite, which reached −5.6%, compared with pure PLA, for corn cob weight fraction 20 wt.%. The hardness of any composite depends on the intermolecular bonds between the filler, corn cob, and the matrix, PLA. Consequently, the enhancement of hardness of PLA-CC composite with weight fraction 10 wt.% corn cob indicates an enhancement in the bonds between the PLA and corn cob due to the well distribution of corn cob inside the PLA. It was proved that the good filler distribution inside the matrix improves the load transfer between them [36]. On the other hand, increasing corn cob weight fraction up to 15 and 20 wt.% might lead to corn cob filler agglomeration, weakening the intermolecular bonds between the PLA and the corn cob filler.

**Figure 9:**
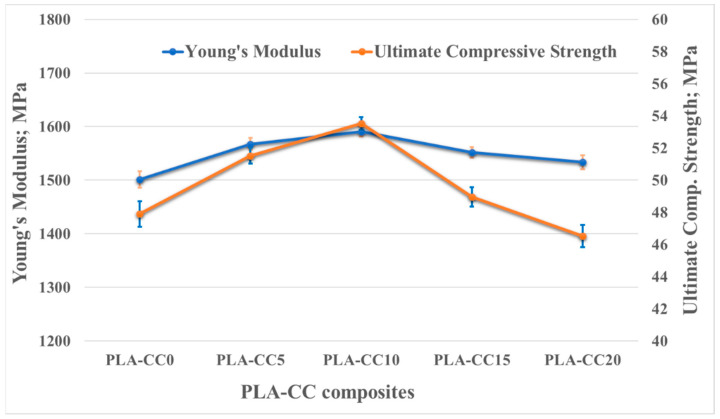
PLA-CC composites ultimate strength and Young’s mdulus for different corn cob weight fractions.

Investigating the load carrying capacity of the PLA-CC composites, a compression test was performed, and the mechanical properties were extracted as shown if Figure 9. Figure 9 illustrates the change in the ultimate compressive strength and Young’s modulus with the change of corn cob weight fraction into the PLA. For both Young’s modulus and ultimate compressive strength, there was an increase in the values due to the addition of corn cob filler up to an optimal value (10 wt.%), beyond which a drop in both of them occurred. The Young’s modulus and ultimate compressive strength of PLA-CC10 were 1590 MPa and 53.5 MPa, respectively, with an enhancement of 6% and 12% with respect to pure PLA. The enhancement in the composite stiffness could interpret as the enhancement in the composite harness [18]. The noticed improvement in the PLA-CC strength may occur because of the uniform distribution of corn cob particles inside the PLA, which facilitates dissipation and absorption of applied compression load. Yang et al. [37] claimed that the existence of filler inside the matrix might stop crack initiation and propagation. However, increasing the corn cob weight fraction to 15 and 20 wt.% decreased the stiffness and strength of the produced PLA-CC composites. The decrease in the mechanical properties for such weight fractions could be attributed to the agglomeration of corn cob filler inside the PLA matrix, weakening the bond between the matrix molecular.

**Figure 10:**
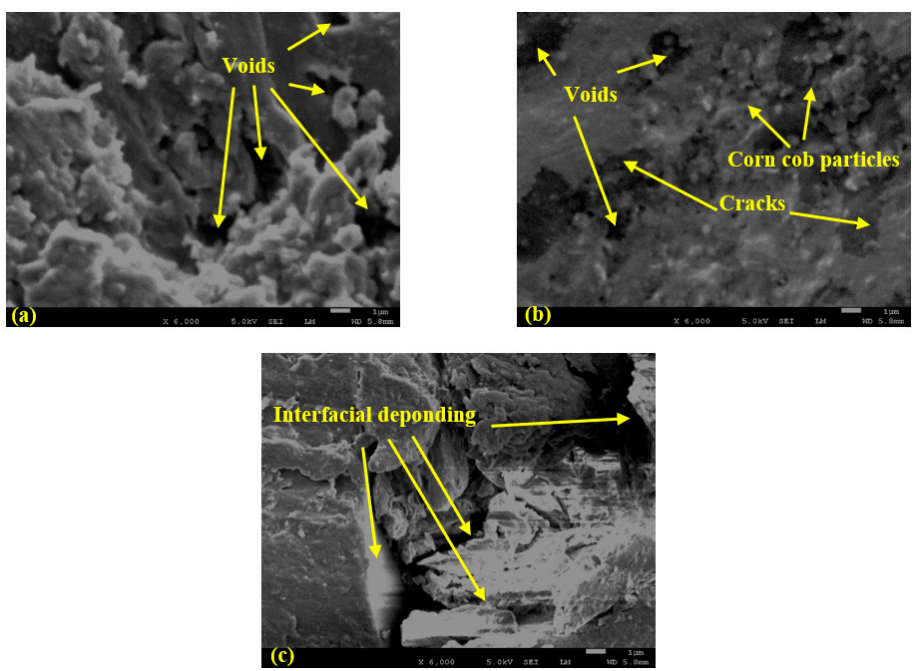
SEM micrographs of the fractographic features of (**a**) PLA, (**b**) PLA-CC10, and (**c**) PLA-CC20.

**Figure 11:**
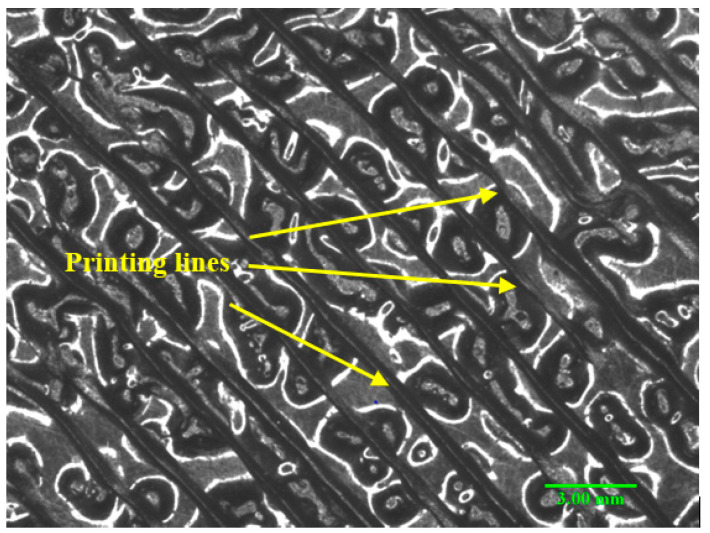
Microscopic image for the surface of pure PLA sample.

The fractures of the pure PLA, PLA-CC10, and PLA-CC20 were evaluated using a scanning electron microscope, as shown in Figure 10. It is clear that the fractographic features are different in the three cases. The fracture morphology of the pure PLA, Figure 10a, shows many voids, which is in line with the results of Abeykoon et al. [38], who found that even the 3D printing setting is tuned to give 100% density, it doesn’t guarantee the production of samples with no voids. Figure 11 illustrates a microscopic photo for the surface of the produced samples before the compression test. It is obvious the sample surface has many voids. The appeared voids can decrease the mechanical properties of the pure PLA sample. The fractographic features of the PLA-CC10, Figure 10b, illustrates the appearance of void but less than PLA-CC0. That occurred because some of the corn cob filler filled such voids, and some corn cob powder appeared on the fractured surface. Furthermore, there are some cracks that was initiated during the compression test. In the case of PLA-CC20, Figure 10c shows that the agglomeration of corn cob particles facilitates interfacial deponding, resulting in the deterioration of the composite mechanical properties.

**Figure 12:**
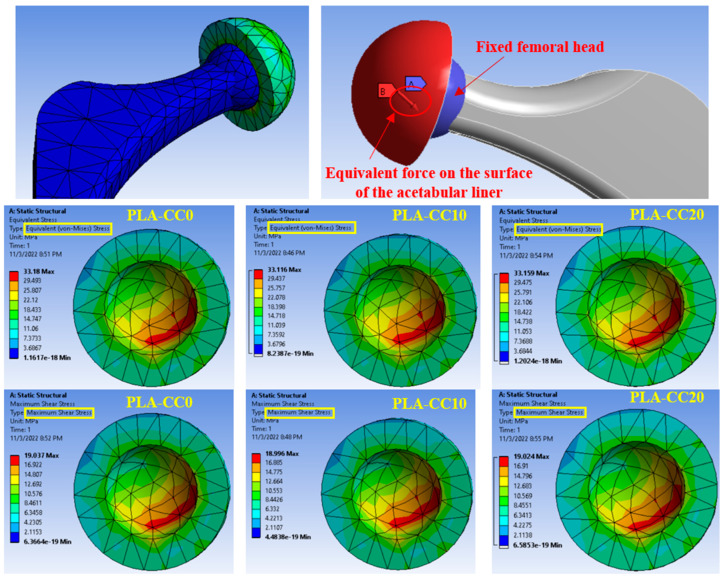
FEM of a hip joint and the different induced stresses.

**Figure 13:**
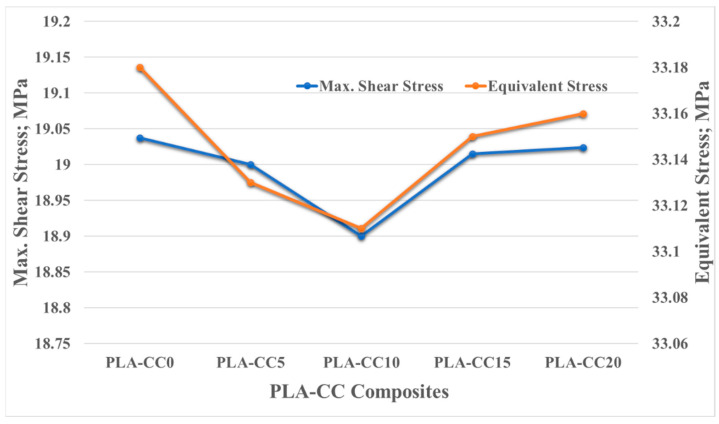
The equivalent and maximum shear stresses on the surface of the articulator liner.

The mechanical evaluation of PLA/corn cob composite shows that incorporating corn cob into PLA can enhance PLA properties. As the developed PLA composite was proposed to be used for artificial implants, a finite element model for a hip joint, as a case study, was built and fed with the extracted PLA-CC properties from the experiments to evaluate the performance of the proposed composite in a real case. Evaluating the generated stresses due to loading can identify the load-carrying capacity of the material. Kuminek et al. [39] claimed that the decrease in the generated contact stresses is evidence of improvement in the load-carrying capacity. Consequently, the stresses generated on the surfaces of the acetabular linear during loading, human weight, were deduced using ANSYS, as shown in Figure 12 and Figure 13. The main boundary conditions that were illustrated in the finite element analysis subsection are illustrated in Figure 11. Furthermore, the stresses generated on the surfaces of the acetabular liner for PLA-CC0, PLA-CC10, and PLA-CC20 are shown in the same figure. The finite element results showed a decrease in the different induced stresses due to adding corn cob filler up to 10 wt.%, as shown in Figure 13. Increasing the corn cob weight fraction above the 10 wt.% increased the induced stresses. The finite element results reflect the change in the mechanical properties that were extracted experimentally. Furthermore, the finite element results prove that 10 wt.% is the optimal weight fraction that can be used with the PLA as it has the best load-carrying capacity. In addition, the maximum generated stress due to loading for 10 wt.% corn cob is 33.11 MPa, less than the ultimate strength of PLA-CC10 by 40%. Such results encourage using the newly developed PLA-CC10 in artificial implants.

**Figure 14:**
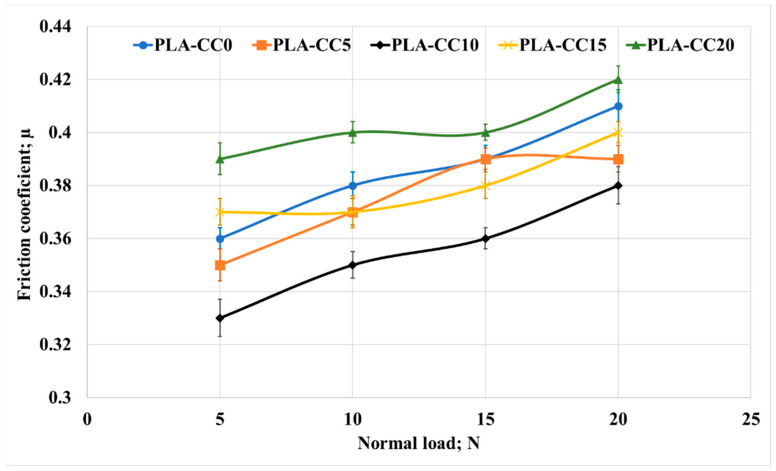
Friction coefficient of PLA-CC composites under different normal loads.

As mentioned before, many implants have motion between their elements which causes wear of the implant parts. Consequently, the effect of corn cob weight fraction on the tribological properties of the PLA composites was investigated. Studying the tribological properties of any material is conducted by exploring the friction coefficient of the material and the specific wear rate [40]. The PLA-CC composites were rubbed under a normal applied load between 5 to 20 N against a stainless-steel counterpart. The friction coefficient average was estimated, as shown in Figure 14. 

Overall, the friction coefficient increased with the increasing of normal load for all composite. Increasing friction coefficient with the normal load occurred due to the heat generated during the friction process that increases with increasing the normal load. Such heat generation could soften the polymeric sample and increase the contact area between rubbed surfaces [41]. In a study by Chang et al. [42], they recorded an increase in the friction coefficient with the increase of the contact temperature, especially at high loads. In addition, it is obvious that PLA-CC10 recorded the lowest friction coefficient among other composites for different loadings. The lowest friction coefficient for PLA-CC10 was 0.33, which is lower than PLA-CC0 (0.36) and PLA-CC20 (0.39) by 9% and18%, respectively.

**Figure 15:**
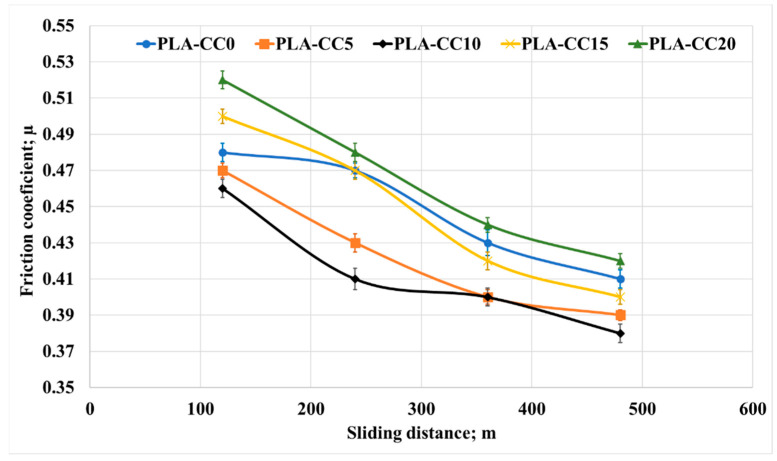
Friction coefficient of PLA-CC composites under different sliding distances.

**Figure 16:**
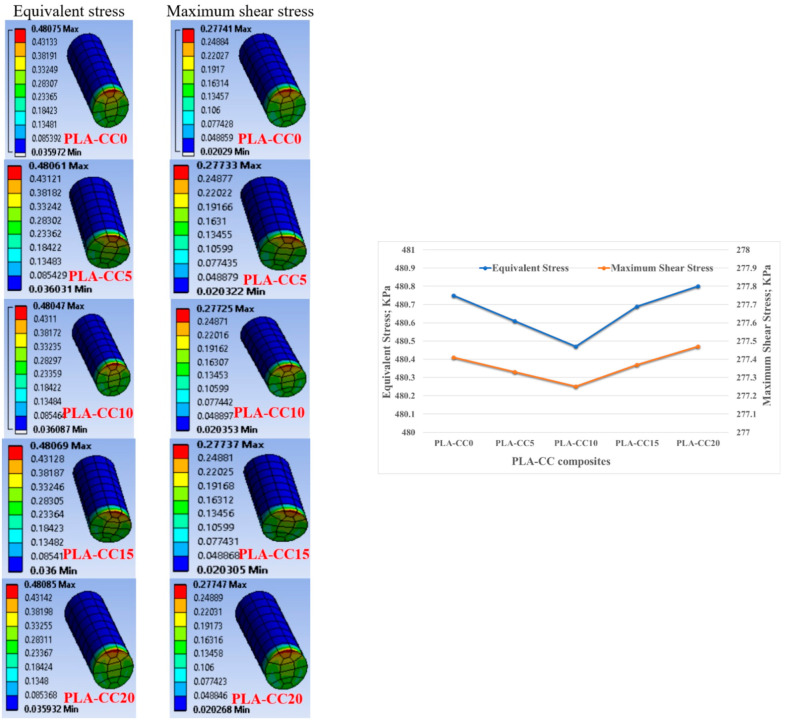
Contact stresses distribution along the PLA-CC composite surfaces.

Usually, biomedical implants are designed to work for long periods. Consequently, they should withstand the load if there is a relative motion between the implant components. Therefore, in the current study, the friction coefficient of PLA-CC composite was investigated under 20 N normal load for different sliding times 5 to 20 min that, represents 120 to 480 m, as shown in Figure 15. Increasing sliding distance led to a decrease in the friction coefficient but with the same behavior for the composites where PLA-CC10 recorded the lowest friction coefficient at different sliding distances. The decrease in the friction coefficient could be attributed to the smoothness in the sample surfaces due to the rubbing for a long distance, and the abrasion occurred against the counterpart. Dass et al. [43] claimed that when polymers rubbed for long periods against steel disks, the generated heat could lead to a localized melting for the polymeric samples; in which a thin film from the polymeric material transmits to the steel counterpart working as a third body between the rubbed surfaces causing a decrease in the friction coefficient. Furthermore, the mechanical properties of the PLA-CC showed an enhancement in the load-carrying capacity, and the enhancement of the load-carrying capacity decreases the friction coefficient of the material [41]. To verify the load-carrying capacity based on the frictional process, the stresses generated during the tribological test were recorded using ANSYS software based on the model in Section 2.3.2. Figure 16 shows the equivalent and maximum shear stresses along the PLA-CC composite samples due to the friction under normal load of 20 N. For all composite samples, the maximum equivalent and shear stress appear at the composite edge where the direction of friction could be identified. Figure 16 illustrates that PLA-CC10 has the lowest contact stresses among other composites, indicating that it has the highest load-carrying capacity. This result is in line with the mechanical properties that showed the highest ultimate strength for PLA-CC10 besides the lowest friction coefficient.

**Figure 17:**
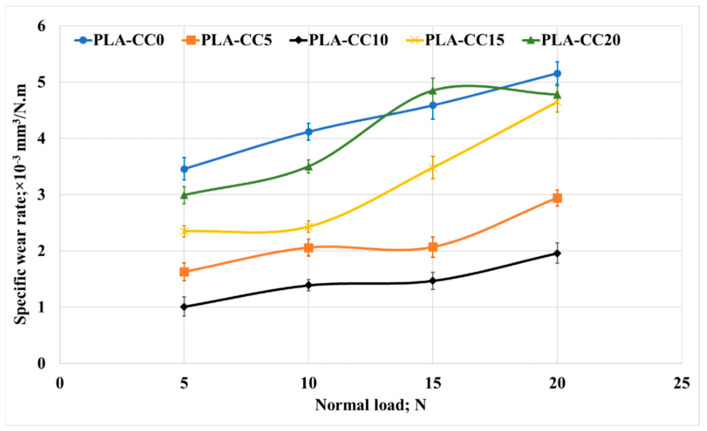
Specific wear rate of PLA-CC composites under different normal loads.

**Figure 18:**
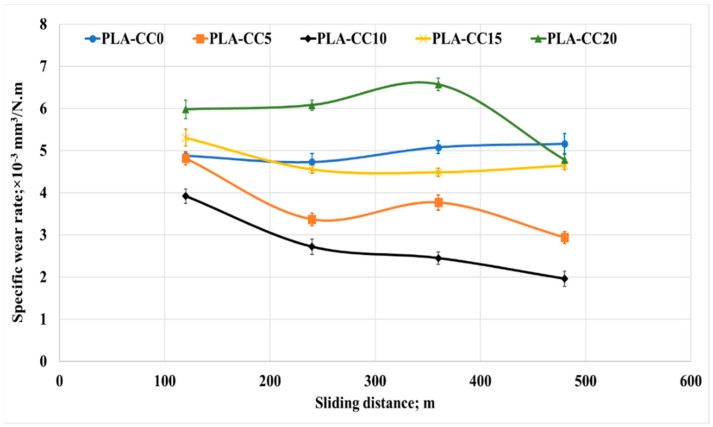
Specific wear rate of PLA-CC composites under different sliding distances.

To investigate the second term in the tribological properties, the specific wear rate was calculated for each PLA-CC composite based on its mass before and after the friction test, density, applied normal load, and sliding distance. Figure 17 shows the change in the specific wear rate for each PLA-CC composite with the change of the applied normal load. Increasing corn cob particle weight fraction by up to 10% enhances the PLA composite’s wear resistance. The enhancement in the wear resistance of PLA-CC10 could be attributed to the enhancement in the compression ultimate strength [44]. In addition, such enhancement in the composite mechanical properties and wear resistance reveals the strong bonding between the PLA matrix and the corn cob particles that prohibited the composite surface from deterioration during the frictional process. Moreover, the maximum shear stress recorded from the finite element model of the frictional process for PLA-CC10 showed the lowest value, reflecting the enhancement in the wear resistance. On the other hand, variation of the applied normal load rises the specific wear rate. As mentioned before, with increasing the normal load, the thermal effect due to the kinetic energy resultant from the rubbing process could transfer layers from the polymeric sample to the disk, which increases the wear rate. In addition, such heat softens the composite surface and increases the shear resistance, increasing the wear rate. In the same manner, the specific wear rate was measured for different sliding distances, as shown in Figure 18. PLA-CC10 still maintains its cohesion even with a change in the sliding distances. However, increasing the sliding distances decreased the specific wear rate for all PLA composites. The decrease in the wear rate could be attributed to two reasons; first, the shear resistance decreased with the increase of the sliding distance due to the smoothness of the surfaces; second, the transferred layers occurred at the beginning of the frictional process acts as a third body and work as a lubricant between the sliding surfaces.

## 4. Conclusions

In conclusion, the current study focuses on developing a new polymer composite that can be used with 3D printers to develop biomedical implants. The following conclusions are drawn:

A complete production methodology for converting natural material, corn cob, from its original nature to a micro filler was conducted.

A PLA/corn cob filament was fabricated with different corn cob weight fractions that suit the utilization with 3D printers.

Increasing corn cob weight fraction decreases the PLA composite density by more than 26%.

Increasing corn cob weight fraction up to 10% led to an enhancement in the PLA composite hardness, Young’s modulus, and ultimate compressive strength by 10%, 6%, and 12%, respectively.

Incorporating corn cob into PLA by up to 10% enhanced the wear resistance of the PLA-CC composite.

The load-carrying capacity was evaluated using finite element analysis for a hip joint as a case study and for the frictional process. In both models, corn cob decreased the contact stresses, reflecting the improvement in the load-carrying capacity.

Eventually, It is recommended to modify the corn cob particles chemically before incorporation inside the PLA to increase the bonding strength between the PLA and corn cob; consequently, more enhancement in the mechanical and tribological properties. Furthermore, a type of heat treatment could be applied to improve the quality of the composite.

## Data Availability

Not applicable.
